# A modified single-endobutton technique combined with nice knot for treatment of Rockwood type III or V acromioclavicular joint dislocation

**DOI:** 10.1186/s12891-021-04915-0

**Published:** 2022-01-03

**Authors:** Fangning Hu, Shumei Han, Fanxiao Liu, Zhuang Wang, Honglei Jia, Fu Wang, Lingfei Hu, Jing Chen, Bomin Wang, Yongliang Yang

**Affiliations:** 1 Department of Orthopaedics, Shandong Provincial Hospital Affiliated to Shandong First Medical University, No. 324, Road Jing Wu Wei Qi, Jinan, 250021 Shandong Province China; 2 Department of Radiotherapy, Shandong Cancer Hospital and Institute, Shandong First Medical University and Shandong Academy of Medical Sciences, Jinan, China; 3 Department of Emergency surgery, Linyi People’s Hospital, Shandong First Medical University and Shandong Academy of Medical Sciences, Linyi, Shandong Province China; 4 Department of Hemodialysis, Linyi People’s Hospital, Shandong First Medical University and Shandong Academy of Medical Sciences, Linyi, Shandong Province China

**Keywords:** Acromioclavicular joint dislocation, Nice knot, Endobutton technique

## Abstract

**Purpose:**

Double-endobutton technique, as a widely accepted strategy for the treatment of acromioclavicular joint dislocation, is undergoing constant improvement. This study aims to assess the clinical effect of a modified single-endobutton combined with the nice knot in the fixation of Rockwood type III or V acromioclavicular joint dislocation.

**Methods:**

From January 2016 to June 2019, 16 adult patients (13 males and 3 females) with Rockwood type III or V acromioclavicular joint dislocation were treated with a modified single-endobutton technique combined with the nice knot in our department. The age ranged from 18 to 64 years old with an average of 32.8 years old. Operative time, intraoperative blood loss, post-operative clinical outcomes and radiographic results were recorded and analyzed. Preoperative and last follow-up scores in the Constant-Murley Scale, Neer score, Rating Scale of the American Shoulder and Elbow Surgeons and VAS scale and complications such as infection, re-dislocation, implant loosening, medical origin fracture and hardware pain were recorded and evaluated.

**Results:**

Sixteen patients were followed up for 6 to 18 months with an average of 10.3 months. The operative time was 50–90 min with an average of (62.5 ± 3.10) min. The intraoperative blood loss was 30–100 ml, with an average of (55.0 ± 4.28) ml. The complications, such as wound infection, internal fixation failure and fractures, were not found in these cases. According to Karlsson criteria, there were excellent in 14 cases, good in 2 cases at the final follow-up. The mean VAS score of the patients was 5.88 ± 0.26 preoperatively, compared with 0.19 ± 0.14 at the final follow-up evaluation. The difference was statistically significant (*P* < 0.05). The mean Constant score was 45.5 ± 2.0 preoperatively, compared to 94.0 ± 0.73 at the final follow-up evaluation. The difference was statistically significant (*P* < 0.05). Patients had statistically significant preoperative and postoperative AC (acromioclavicular distance) and CC (coracoclavicular distance) distances (*P* < 0.05); 6 months postoperatively the AC(*P* = 0.412) and CC(*P* = 0.324) distances were not statistically significant compared to the healthy side.

**Conclusion:**

Nice knot provides a reliable fixation for the single-endobutton technique in the treatment of acromioclavicular dislocations. The modified single-endobutton technique combined with the nice knot can achieve good clinical outcomes in the treatment of Rockwood type III or V acromioclavicular joint dislocation.

## Introduction

The acromioclavicular joint is a complex linkage joint consisting of the acromioclavicular joint surface and the acromioclavicular ligament, rostral ligament and joint capsule, which anchors the clavicle to the scapula. The movement of the scapula is extremely complex and therefore treatment of a scaphoid injury can be very difficult [1]. Acromioclavicular joint dislocation is a common type of acromioclavicular joint injury, accounting for approximately 9% of shoulder injuries. It is most commonly seen in shoulder sports injuries and is mainly caused by impingement on the shoulder crest when the upper arm is in an inversion position [2]. The Rockwood staging system is often used for acromioclavicular dislocations and is divided into types I-VI [3]. For Rockwood type I-II injuries with a small dislocation of the acromioclavicular joint, conservative treatment are possible. Rockwood IV-VI is recommended for surgery due to the severity of the injury and the large displacement of the acromioclavicular joint, however, the treatment of Rockwood type III injuries is still controversial [4, 5]. Several studies [6] indicated that surgical treatment does not improve the general health status of patients compared to non-surgical treatment. Meanwhile, numerous studies [7] have shown that Rockwood III can be treated surgically to restore the normal anatomy of the acromioclavicular joint, re-establish joint stability and achieve good functional outcomes. Therefore, for young patients with high activity levels, surgery is recommended [3]. The most commonly used surgical procedure is an internal fixation with the double Endobutton technique [8–11]. However, the procedure can be complicated by collar plates trapped in the bone channel, fractures of the rostral process or clavicle, and loosening of the knot after the operation [12].

**Fig. 1:**
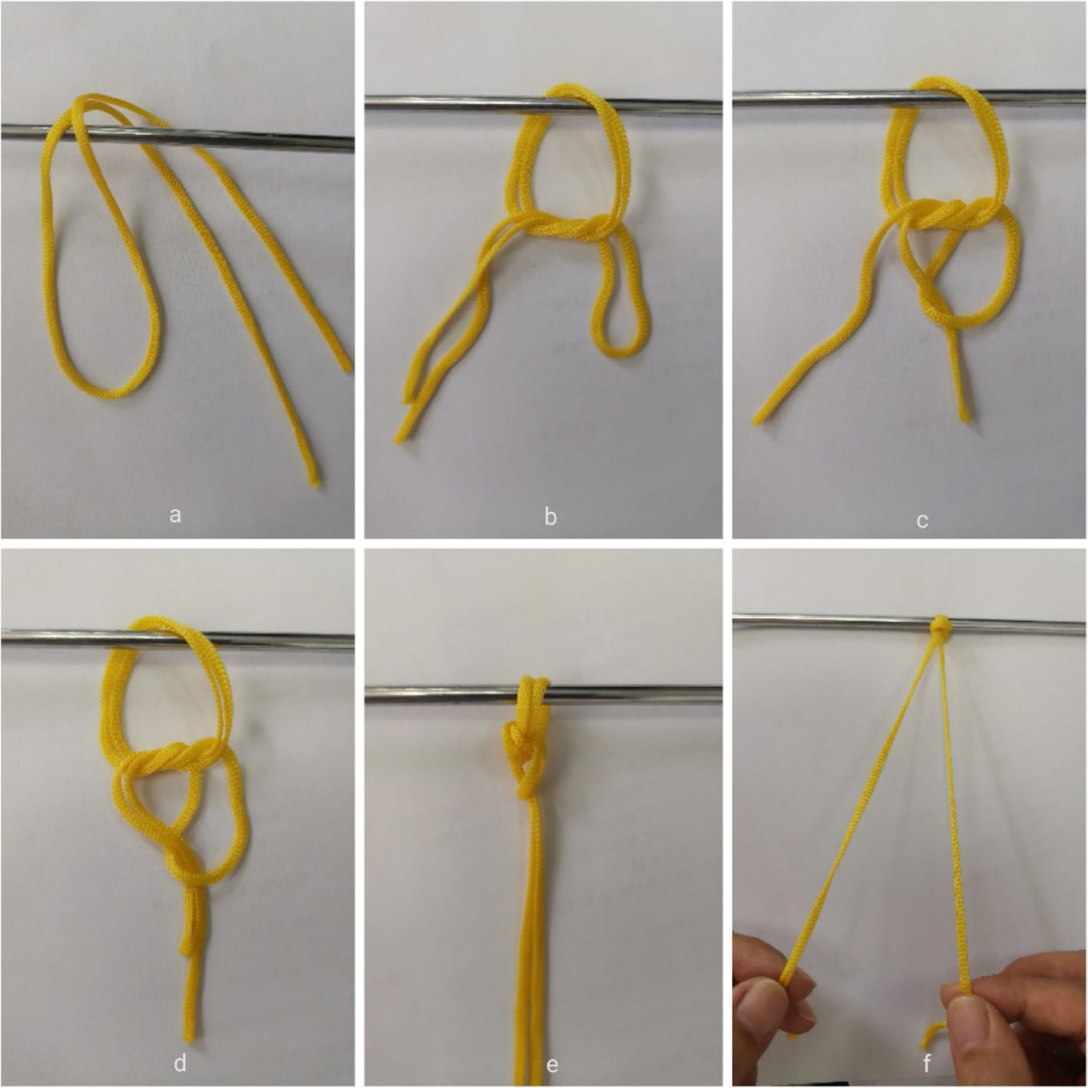
Nice Knot technique. **a** A double-over suture is passed around the tissue. **b** A single square knot is thrown. **c**, **d** The 1/2 free limbs are passed through the loop. **e** The knot is dressed. **f** The knot is slid down by pulling the 2 free limbs apart and the tightened knot is ready to be secured with 3 alternating half-hitches or a surgeon’s knot

Pascal Boileau [13] described a new, simple and effective self-locking sliding two-wire knot (Fig. 1). And instead of wire cables, a nice knot is used to fix tendons, ligaments, fractures, etc. Two studies [14, 15] also showed that the nice knot had good biomechanical properties. In fact, the nice knot has been reported to be superior for comminuted patellar fractures, clavicle fractures and for closure of small and medium-sized trauma [16–20]. Recently, the nice knot has been used in combination with the double endobutton to treat acromioclavicular dislocations with good clinical results [21].

Therefore, we have modified the double endobutton technique by replacing the double collar with a single collar plate and wrapping it around the rostral process, combined with nice knot internal fixation for acute acromioclavicular dislocation. This study investigates the clinical efficacy of the nice knot-assisted modified single-endobutton technique in the treatment of acute Rockwood III or V acromioclavicular dislocation.

## Materials and methods

The study was approved by our ethics committee and all patients gave their informed consent and signed the informed consent form.

### Patient selection

Patient inclusion criteria (1) Closed unilateral Rockwood III or V subluxation of the acromioclavicular joint; (2) Age ≥ 18 years; (3) Normal shoulder function before the injury; (4) Patient follows our treatment plan. Patient exclusion criteria: (1) Chronic acromioclavicular joint dislocation; (2) Patients with open injuries or vascular or nerve damage; (3) Age < 18 years; (4) Patients with severe osteoporosis; (5) Patients with co-morbidities that cannot tolerate surgery; (6) Patients who refuse surgical treatment; (7) chronic acromioclavicular joint dislocation (more than 3 weeks after the injury).

Review of 15 (12 Rockwood III and 3 V) consecutive patients who had unilateral Rockwood III or V acromioclavicular dislocations and 1 patient with Cho type IIC distal clavicle fracture [22] treated with modified single-endobutton technique combined with the nice knot at our hospital from January 2016 to June 2019. All patients had a closed injury and were admitted to the hospital with a positive X-ray of both shoulders and a lateral X-ray, CT and MRI of the affected shoulder to clarify the diagnosis and improve the preoperative examination. All were treated with a modified single-endobutton collar plate technique combined with nice knot surgery, with intraoperative and postoperative X-ray fluoroscopy. Regular post-operative follow-up was performed.

### Operative technique

**Fig. 2:**
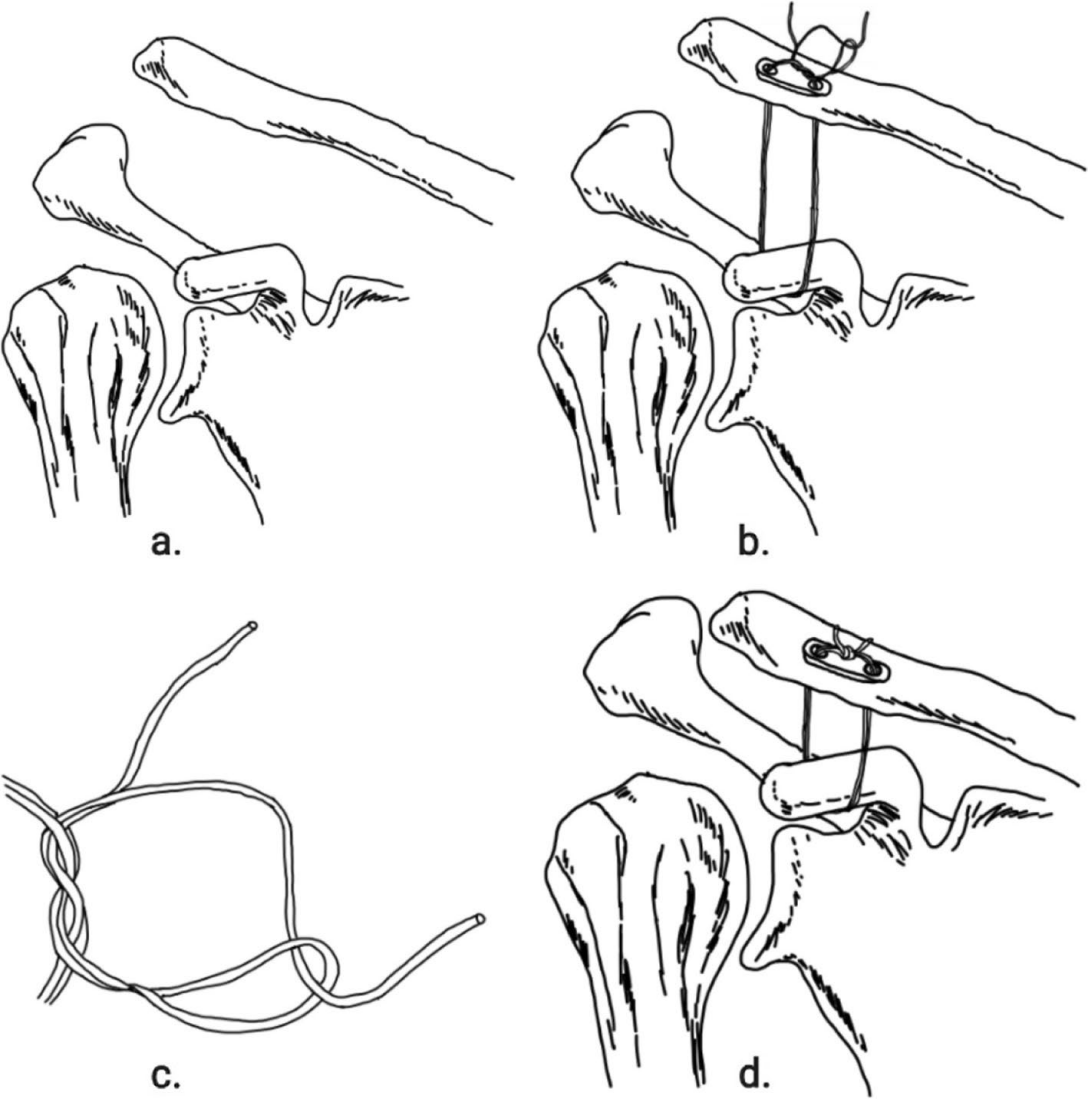
Illustration of a modified single-endobutton technique combined with the nice knot for treatment of Rockwood type IIIACD. **a** Rockwood type III or V acromioclavicular joint dislocation. **b** The two strands of Endobutton suture are folded into four strands and introduced through the medial hole of the clavicle, around the rostral process and leading out at the lateral hole of the clavicle, respectively out of the hole of Endobutton. The acromioclavicular joint is repositioned and fixed on the Endobutton using the nice knot and gradually tightened. **c** Illustration of a nice knot. **d** If the joint was repositioned, at least three single knots were added to the nice knot to strengthen the fixation

All operations are performed by the same surgeon team. The patient is under general anesthesia and in the beach chair position. A longitudinal incision of approximately 3 cm is then made vertically upwards around the coracoid to reveal the coracoid process conjunctive tendon and coracoid root. The injured limb is internally rotated in the inward position, and a traction wire is placed along the base of the coracoid from inward to outward around the coracoid and into a reserve. Bone channels of 2 mm each drilled 1.5 cm and 3 cm distal to the acromioclavicular joint and 2 cm anterior to the posterior border of the clavicle. The traction wire is placed in the bone channel and reserved for use. The double-endobutton is split and keep only the single-endobutton. The two strands of endobutton suture are folded into four strands and introduced through the medial hole of the clavicle, around the rostral process and leading out at the lateral hole of the clavicle, respectively out of the hole of endobutton. The acromioclavicular joint is repositioned and fixed on the endobutton using the nice knot and gradually tightened. Intraoperatively, the acromioclavicular joint was observed with C-arm fluoroscopy. If the joint was repositioned, at least three single knots were added to the nice knot to strengthen the fixation (Fig. 2).

### Pre- and post-operative management

**Fig. 3:**
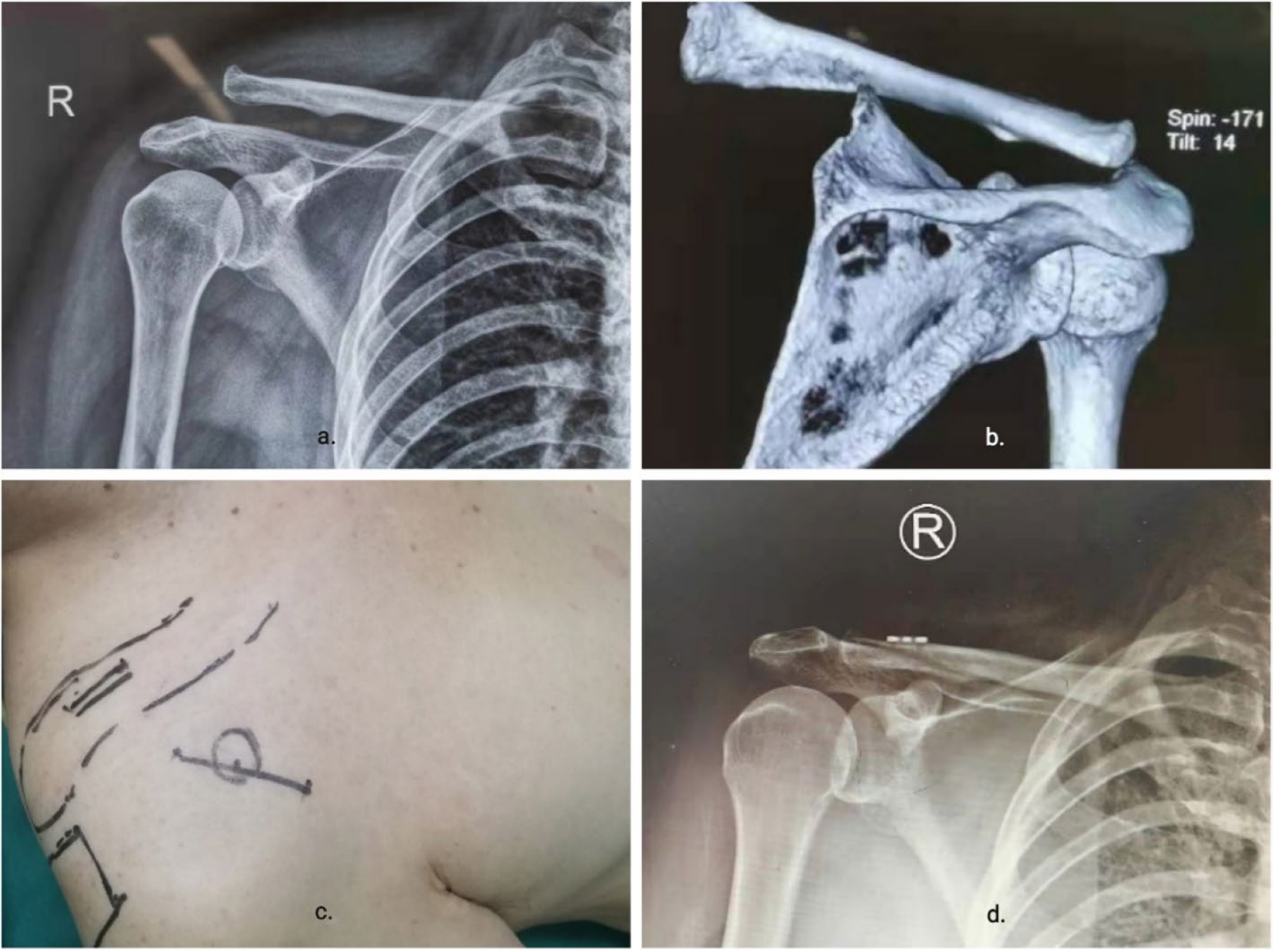
Introduction to the perioperative period. **a**, **b** Pre-operative X-ray and CT evaluation and measurement of acromioclavicular joint dislocation. **c** Primary body surface location and marking by imaging and anatomical position. **d** Postoperative imaging assessment of reduction

**Fig. 4:**
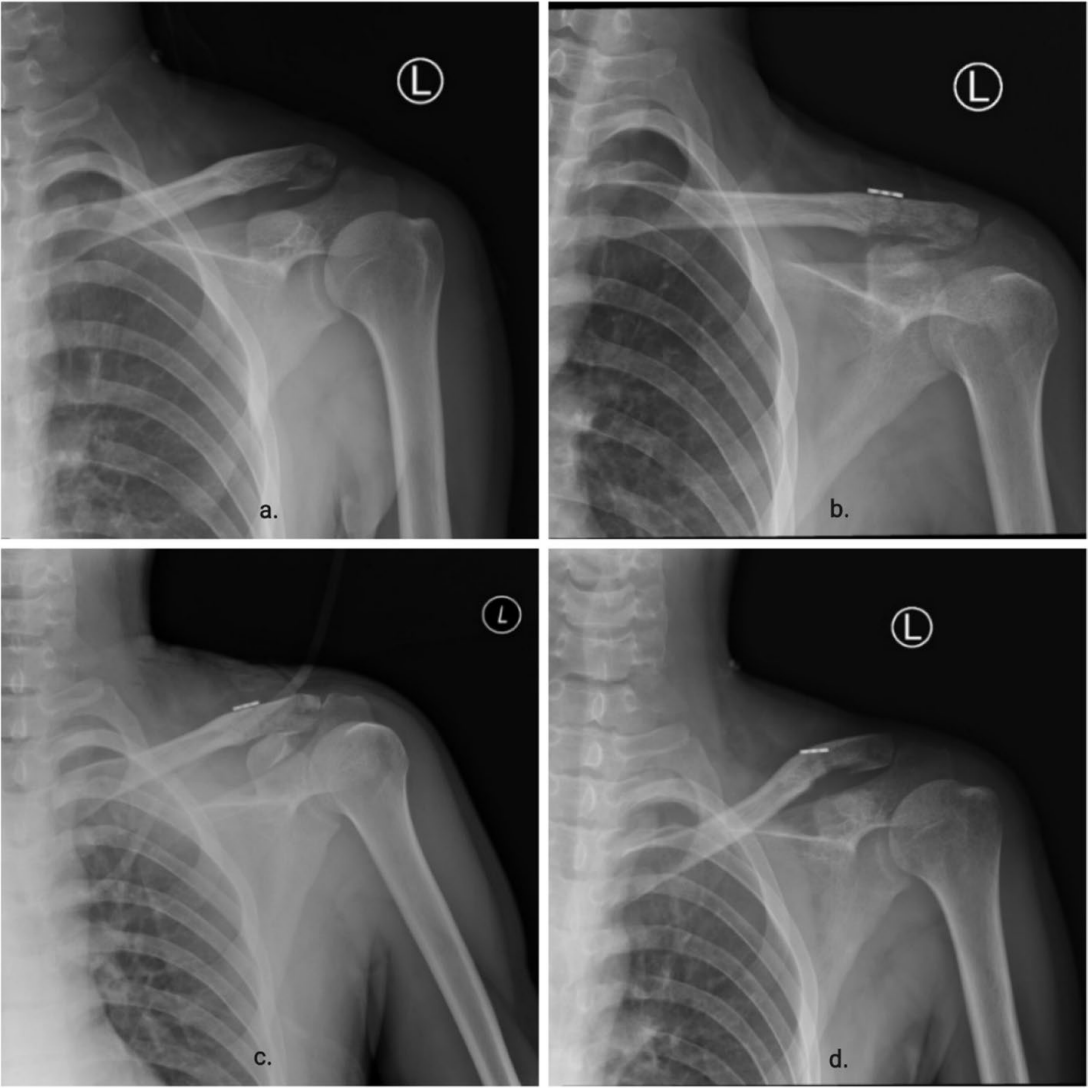
Imaging follow-up of patients. **a** Pre-operative X-ray showed dislocation of the acromioclavicular joint with distal clavicle fracture. **b** X-rays on the second postoperative day showed a good dislocation of the acromioclavicular joint and a fracture of the distal clavicle. **c** X-ray 40 days after surgery shows bone scab formation and fracture lines are blurred. **d** Postoperative x-ray at 3 months showed almost healing of the distal clavicle fracture and good repositioning of the acromioclavicular joint

Pre-operative imaging is performed to measure and mark the distance from the clavicle to the coracoid on both sides of the patient and the distance from the base of the coracoid to the acromioclavicular joint on X-ray. The patient is well under anesthesia and initially positioned to mark the incision location. Post-operative fluoroscopy showed that the acromioclavicular joint was well repositioned (Fig. 3). The surgical time (min) was recorded as the time from skin incision to the closure of the wound. Intraoperative blood loss (ml), fluoroscopy time(s) and postoperative complications as well were recorded. All patients had X-rays taken the day after surgery and were given functional exercises in a forearm sling suspension. Active forward flexion, extension and abduction of the shoulder joint started 1 week after surgery. Partial weight-bearing on the shoulder joint started 2 months after surgery. Full weight-bearing was allowed 3–6 months after surgery. Patients were followed up regularly at 3 weeks, 6 weeks, 3 months, 6 months and 12 months after surgery to guide rehabilitation and functional exercises. We had also found good application of this technique to the Cho type IIC distal clavicle fracture. (Fig. 4). At preoperative and final follow-up, patients’ shoulder function was evaluated according to the Visual Analgesia Score (VAS), Neer score, American Shoulder and Elbow Surgeon Scale (ASE), and Constant-Murley score. The acromioclavicular (AC) distance is measured between the stop of the tapered ligament and the distal clavicle [23]. The coracoclavicular (CC) distance is measured between the uppermost border of the coracoid process and the opposing clavicular surface [24].

### Statistical analysis

All data were analyzed using IBM SPSS Statistics 23.0 statistical software for analysis. Parametric data, such as operative time, fluoroscopy time and blood loss, are described as mean ± standard deviation (SD). Pearson’s chi-square test and Fisher’s exact test for comparing classification results. A paired t-test was used to compare post-operative and pre-operative functional scores. Statistical significance level was set at *p*-value ≤0.05.

## Results

Of the 15 patients included with Rockwood III or V acromioclavicular dislocation and 1 patient with Cho type IIC distal clavicle fracture, 13 were male patients and 3 were female patients. The patients’ ages ranged from 18 to 65 years with a mean of (36.7 ± 12.7); five of them had a history of smoking and alcohol; statistics showed that six were left-sided and ten were right-sided. The mechanism of injury was traffic injury in 9 cases, direct violence in 1 case and a fall in 6 cases. The patient’s BMI was 23.77 ± 3.03 (range 18.1–28.6); the anesthesia record sheet showed operation time was 62.5 ± 12.4 min, (range 50–90 min); intraoperative bleeding was 55.0 ± 17.1 ml (range 30–100) ml. The patient’s time in the hospital was 5.8 ± 0.8 days (5-7d). (Tables 1 and 2).

**Table 1:** Patient demographics

Variable	
No. of cases	16
Gender (M/F)	12/4
Age (Year, mean ± SD)	36.7 ± 12.7
BMI (mean ± SD)	23.77 ± 3.03
History of tobacco and alcohol (Y/N)	5/11
Left or Right (L/R)	6/10
Injury mechanism	
Traffic accidents	9/16
Direct violent injury	1/16
Fall	6/16
Follow-up time (month, mean ± SD)	13.1 ± 2.3

*M* Male, *F* Female, *BMI* Body Mass Index, *SD* Standard deviation

**Table 2:** Operation related factors

Variable	
Operative time (min, mean ± SD)	62.5 ± 12.4
Blood loss (ml, mean ± SD)	55.0 ± 17.1
Fluoroscopy time (s, mean ± SD)	8.6 ± 1.7
Hospitalization days (d, mean ± SD)	5.8 ± 0.8

*SD* Standard deviation

All patients received 13.1 ± 2.3 (range 6–18) months of follow-up. There were no complications such as infection or vascular or neurological damage, no cases of rostral or clavicle fractures, and no loosening or breaking of internal fixations.

We have graded them on the Karlsson scale. The shoulder joint was well repositioned at the final follow-up, with 14 excellent cases and 2 good cases, representing an excellent rate of 100%. The preoperative VAS score was 5.9 ± 1.0 (range 4–8) and the VAS score at the last follow-up was 0.3 ± 0.6 (range 0–2), with a statistically significant difference (*P* < 0.05). The preoperative Constant-Murley score was 45.5 ± 8.0 (range 30–56) and the Constant-Murley score at the last follow-up was 93.8 ± 2.3 (range 87–98), a statistically significant difference (*P* < 0.05). The American Shoulder and Elbow Surgeon Score (ASES) at the last follow-up was 94.8 ± 1.9 (range 92–99), which was significantly higher than the preoperative ASES of 46.2 ± 4.9 (range 39–53) (*P* < 0.05). The final follow-up Neer score was 94.1 ± 2.1 (range 90–98), a significant increase from the preoperative Neer score of 46.3 ± 5.3 (range 38–54) (*P* < 0.05). (Table 3).

**Table 3:** Postoperative follow-up results

Group	Pre-op	Post-op	*P*-value
ASES (mean ± SD)	46.2 ± 4.9	94.8 ± 1.9	<0.05
Neer score (mean ± SD)	46.3 ± 5.3	94.1 ± 2.1	<0.05
Constant-Murley score (mean ± SD)	45.5 ± 8.0	93.8 ± 2.3	<0.05
VAS (mean ± SD)	5.9 ± 1.0	0.3 ± 0.6	<0.05

*VAS* Visual analogue scale, *ASES* American Shoulder and Elbow Surgeons’ Form, *SD* Standard deviation

Removing a patient with a distal clavicle fracture, we measured the AC and CC distances on the healthy side of the patient, preoperatively, postoperatively, 3 and 6 months postoperatively on x-rays of 12 patients with type III and 3 patients with type V acromioclavicular dislocations. Significant reduction in patient’s post-operative AC compared to pre-operative. Post-operative (3.05 ± 0.30) AC was significantly reduced in patients compared to pre-operative (12.70 ± 1.76), with a statistically significant difference (*P* < 0.05). Post-operative (8.75 ± 0.34) CC was statistically significantly smaller than preoperative (16.81 ± 2.86), with a statistically significant difference (P < 0.05). The AC distance at the 6-month postoperative follow-up was 3.25 ± 0.27 (range 2.7–3.6) and 3.16 ± 0.30 (range 2.5–3.6) on the healthy side, with no statistically significant difference (*P* = 0.412). The CC distance at the 6-month postoperative follow-up was 8.95 ± 0.40 (range 8.2–9.4) and 8.80 ± 0.37 (range 8.1–9.3) on the healthy side, with no statistically significant difference (*P* = 0.324). (Table 4).

**Table 4:** Imaging evaluation

Variable	Pre-op	Post-op	3 M	6 M	HS	P1	P2
AC (mm, mean ± SD)	12.70 ± 1.76	3.05 ± 0.30	3.23 ± 0.29	3.25 ± 0.27	3.16 ± 0.30	<0.05	0.412
CC (mm, mean ± SD)	16.81 ± 2.86	8.75 ± 0.34	8.93 ± 0.40	8.95 ± 0.40	8.80 ± 0.37	<0.05	0.324

*AC* AC-distance, *CC* CC-distance, *HS* Healthy side, *P1* Pre-Post, *P2* 6 M-HS

## Discussion

The Nice knot has advantages such as sliding locking, easy operation and reliable fixing power. Boileau [13] reports on the versatility of the nice knot for arthroscopic repair of tendon and ligament injuries, for binding post-osteotomy fractures and for repositioning and fixing butterfly fractures in fractures, as well as for repairing the separation of the inferior tibiofibular joint in fixation. Mengcun Chen [20] applied a nice knot to comminuted patella fracture and achieved good clinical results. P Collin [14] analyzed the biomechanics of the nice knot in the repair of rotator cuff injuries and concluded that the knot provided a good sliding lock and significantly reduced the risk of knot elongation during dynamic strain. On the application of a nice knot in acromioclavicular dislocation, Zhongxing Ma [21] proposed a modified double endobutton technique in combination with Nice knot for the treatment of Rockwood III orV acromioclavicular dislocation and achieved good clinical results. We have modified the endobutton technique by replacing the double-endobutton with a single-endobutton and using the nice knot to treat Rockwood III or V acromioclavicular dislocations, making it easier and more convenient to reposition the acromioclavicular dislocation intraoperatively.

There are various surgical options for dislocation of the acromioclavicular joint, such as fixation with pins, screws or plates, as well as reconstruction of the rostral or acromioclavicular ligaments or distal clavicle resection. More complications were associated with internal fixation of the acromioclavicular joint dislocation with Clinique pins [25, 26]. The most commonly used surgical procedures are the clavicle hook plate fixation [27], the Endobutton technique [21, 28, 29] and the Tightrope [30, 31] coracoid collateral ligament repair and reconstruction.

Clavicle hook plate internal fixation is the current treatment for acromioclavicular joint dislocation [27]. The clavicle hook plate provides mechanical stability in the longitudinal and horizontal directions. However, there is a high incidence of complications, including postoperative shoulder pain, limited shoulder movement, foreign body sensation, rotator cuff injury, acromion impingement syndrome, osteolysis of the acromion, distal clavicle fracture and plate fracture [32–37]. In addition, the patient will need to remove the internal fixation device a second time.

The double-endobutton technique for the treatment of acromioclavicular dislocations was first reported by Struhl [29] in 2007 and has since been widely used. Endobutton system consisting of titanium plates, coils and sutures. During the operation, the acromioclavicular joint was repositioned and temporarily fixed with a Kirschner pin. The base of the coracoid process and clavicle were drilled successively. The two titanium plates of the endobutton are fixed by coils above the clavicle and below the coracoid. The reconstruction of the conical and trapezius ligaments was achieved by drilling holes in various parts of the distal clavicle. The double endobutton technique significantly improves the patient’s early shoulder pain, range of motion in shoulder abduction supination and forward flexion supination. However, biomechanical studies [38] have shown that the endobutton technique fails mainly because the strength of the coils is greater than the strength of the bone under overload conditions, resulting in bone damage. The main complications of this technique are fractures of the clavicle or rostral process, loss of repositioning, calcification of the rostral-clavicular ligament and traumatic acromioclavicular arthritis [38–40]. In addition, the endobutton technique only limits the up and down displacement of the clavicle and lacks horizontal stability [41].

The Tightrope technique is a new system that has emerged in recent years to reconstruct the rostro-clavicular ligament and can be considered an improved upgrade to the endobutton system. It combines the advantages of the endobutton system and can be adjusted to the length of the coil as required. Studies [38, 42] have shown that the Tightrope technique offers significant advantages in terms of stability and postoperative clinical outcomes. However, it is still at risk of acromion fracture [43].

According to the characteristics of endobutton technology, we improve the technology in the following aspects: (1) Intraoperative 4.5 mm bone tracts are no longer used. We believe that the 4.5 mm bone channel is too large for the plate to sink into the bone channel. Intraoperative positioning at the central base of the coracoid is particularly difficult, especially in the absence of arthroscopic surveillance, and if the position is shifted intraoperatively, the fracture at the base of the coracoid is easily fractured and fixation fails [12, 44]. Two 2 mm channels are drilled in the distal clavicle at 1.5 cm and 3 cm medially, the coil is passed through the channel and around the base of the coracoid, and the plate is placed on the clavicle and fixed with a nice knot. This not only reduces the risk of fracture from drilling the rostral process but also reduces the diameter of the process and prevents the plate from sinking into the bone. The surgical incision is minor and does not have to rely on arthroscopic assistance; (2) We drill two bone channels in the clavicle, which is equivalent to reconstructing the vertebral and oblique ligaments at the same time, without additional operations; (3) After the dislocation of the acromioclavicular joint was reduced intraoperatively, the clavicle was fixed with Nice Knot. The main feature of the nice Knot is that it can be slid and locked. The knot can be tightened during surgery and then the acromioclavicular joint can be reduced by fluoroscopy. In this group, we treated 15 patients with Rockwood III or V acromioclavicular dislocation and 1 patient with Cho type IIC distal clavicle fracture using the nice knot combined with the modified endobutton technique. No complications such as coracoid fracture, re-dislocation of the acromioclavicular joint or vascular nerve injury were observed after surgery. Post-operative follow-up showed a significant improvement in the function of the affected shoulder compared to the pre-operative period. In the treatment of Rockwood III or V acromioclavicular dislocation with the nice knot assisted modified endobutton technique, we believe that attention should be made to the following: (1) This technique is not recommended for elderly patients with osteoporosis as there is a risk of fracture of the coronoid due to wire loop cutting; (2) The technique involves wrapping the thread around the base of the coracoid. The limb should be placed in the medial position and the anterior ring introduced against the base of the coracoid and the underlying bone to avoid damage to the brachial plexus nerve and the axillary sheath below the coracoid process; (3) The soft tissues around the acromioclavicular joint and clavicular area scar within 4–6 weeks after surgery. Therefore, a forearm sling should be used for 4 weeks postoperatively to avoid excessive abduction leading to loss of reduction.

Although our data showed good clinical results in terms of operative time, intraoperative fluoroscopy time, intraoperative bleeding, and satisfaction with repositioning, there are still some shortcomings in this study: (1) This study is a single-center retrospective clinical case analysis with a low level of evidence and a small number of cases, and a multi-center, large sample case analysis is required to confirm the results of this study. The practicality and feasibility of the nice knot combined with the modified single-endobutton technique for Rockwood III or V acromioclavicular dislocations need to be studied in a large sample; (2) The variables in this study are not unique and there is some selection bias; (3) Although the clinical and imaging results of nice knot are good, more long-term follow-up results are needed to determine whether there are long-term complications. As there was no postoperative CT in this study, it was not possible to accurately assess the horizontal or rational instability.

## Conclusion

In conclusion, the nice knot combined with the modified single-endobutton technique for Rockwood III or V acromioclavicular joint dislocations or Cho type IIC distal clavicle fracture is an option for the treatment of acromioclavicular dislocations as it makes intraoperative reduction easier has significant clinical efficacy, improves the function of the shoulder joint, reduces pain and reduces complications. We will further expand the sample size and extend the follow-up period in future work in order to better improve the technique.

## Data Availability

All data generated or analyzed during this study are included in this published article.
